# When too much closeness harms: circumflex artery injury during mitral valve surgery

**DOI:** 10.3389/fcvm.2023.1183182

**Published:** 2023-10-27

**Authors:** Christian Dumps, Philipp Simon, Evaldas Girdauskas, Felix Girrbach

**Affiliations:** ^1^ Department of Anesthesiology and Surgical Intensive Care Medicine, University Hospital Augsburg, Augsburg, Germany; ^2^ Department of Cardiothoracic Surgery, University Hospital Augsburg, Augsburg, Germany

**Keywords:** mitral valve, cardiac surgery, minimally invasive surgery, valvular annuloplasty, heart valve prosthesis implantation, mitral valve insufficiency, coronary occlusion

## Abstract

Occlusion of the left coronary circumflex artery (LCX) during surgical procedures of the mitral valve is an infrequent but potentially life-threatening complication (1–3). Due to its close anatomical relationship to the posterior mitral valve annulus, there is a relevant risk of causing a stenosis or an occlusion of the left circumflex artery, especially by surgical annular sutures. The perioperative clinical course is heterogeneous, ranging from—initially—asymptomatic or solely electrocardiographic abnormalities to cardiogenic shock. Both severely impaired ventricular contractility or malignant arrhythmia may potentially lead to a weaning failure from cardiopulmonary bypass (CPB) and eventually result in chronic heart failure with persistently reduced ejection fraction. Possible therapeutic strategies include the immediate reopening of causal sutures, aortocoronary bypass grafting or percutaneous coronary intervention (PCI), yet PCI seems to be the preferred method at present.

## 1. Introduction

Surgical repair of severe mitral valve (MV) regurgitation is preferred to MV replacement whenever possible (4, 5). MV repair as compared to MV replacement is associated with reduced perioperative mortality, lower incidence of valve-related complications, improved long-term survival and better postoperative left ventricular function. Additionally, there is no need for long-term anticoagulation (4, 6). Possible complications of the procedure are atrioventricular block due to injury of the His bundle, distortion of the non-coronary or left-coronary aortic valve cusp with subsequent aortic valve insufficiency, residual/recurrent mitral regurgitation as well as a mostly momentous lesion of the LCX. Numerous case reports address the recurring question of the best therapeutic strategy in the case of LCX injury. In the literature, the total cumulative risk of coronary occlusion during MV surgery is reported to be around 0.15%–2.2%, even in high-volume centers (3, 7–9). However, although predisposing factors for LCX injury have been identified and few well-considered and promising preventive approaches have been reported, preoperative risk evaluation for this devastating complication is still insufficient. The following narrative review intends to provide a brief overview of the incidence of LCX injury during MV repair, predisposing factors and possible prevention strategies. Subsequently, an outlook on possible therapeutic approaches is given.

## 2. Anatomical relationship of the LCX to the mitral valve and its implications on LCX injury

The left main coronary artery branches into the left anterior descending artery and the left circumflex artery (LCX). Running along with the left atrioventricular junction in the coronary sulcus, the LCX approaches the posterior part of the mitral valve annulus, especially the P1 area and partially the adjacent subarea of P2. Annular sutures during both MV reconstruction and MV replacement can distort the LCX. It is common sense that the smaller the distance of the LCX to the mitral valve annulus, the higher the risk of LCX injury when performing annular sutures. The role of the coronary dominance pattern with regard to the risk of LCX injury is however less clear and still a matter of debate (1).

Several studies suggest a correlation between the coronary dominance pattern and the distance of the proximal third of the LCX to the mitral valve annulus. Virmani et al. already reported in the 1980s that the mean distance of the mitral valve annulus to the LCX in left dominant coronary artery anatomy was 4.1 mm (range 3–6.5 mm). For codominant suppliers, the mean distance could be measured of 5.5 mm (range 4.5–7.5 mm) and in right dominance 8.4 mm (range 6–11.5 mm) (10). These results were confirmed by an anatomical study on 15 cadaveric specimens by Cornu et al. thirteen years later which shows similar measurement results (11). Kaklikkaya and Yeginoglu (12) also found a correlation between coronary dominance pattern of the heart and proximity of the MV to the LCX, but the distances were on average lower (2.3 mm in left dominance, 3.0 mm in codominant systems, and 5.1 mm in right dominance). In a CT based study from Caruso et al. (13), the distance between the MV annulus was significantly smaller in patients with left dominance compared to right dominance, but there was no statistical difference between patients with left dominance pattern and patients with a balanced pattern. However, they found a significant correlation between the diameter of the LCX and high-risk anatomy (distance between MV annulus and LCX < 3 mm). Accordingly, Kishimoto et al. also found a left coronary dominance pattern to be significant factor affecting minimum LCX to MV annular distance in their recently published, CT-based study (14).

As opposite, a cadaver study from Brazil and study population with an overrepresentation of right coronary artery dominance (81.17%) demonstrated a mean distance of 4.0 **± **1.8 mm for them, 3.6 **± **1.6 mm in specimen with balanced type, and a distance of 2.8 **±** 1.3 mm in 2 cases with left dominant pattern. Based on this data, no statistical difference in the MV to LCX distance could be detected between right and balanced coronary dominance pattern (15). Remarkably, the minimum distance measured between the P1 segment and the LCX was only 1.0 mm in a patient with right dominant anatomy (15). In a perioperative transesophageal echocardiographic assessment by Ender at al. including 110 cases, the nearest distance for the right and balanced type of coronary circulation was measured with 1.3 mm compared to 2.2 mm for the left dominance type, rather underlining the results of Pessa et al. (1). Likewise, Miura et al. did not find a significant correlation between the dominance pattern and the shortest distance to the MV annulus (16). In addition, there are also numerous case reports of patients with an LCX originating directly from the right coronary cusp of the aorta (17–21). Angelini et al. describe the frequency of this anomaly as 0.67% in a cohort including 1,950 patients (22). This anomaly may therefore predispose to LCX injury during MV surgery.

In addition to the individual anatomical predisposition to LCX occlusion, it should also be mentioned that minimal invasive surgical approaches are at least suspected to increase the risk independently. compared with a conventional full sternotomy (23, 24). It should not be forgotten that the minimally invasive technique of mitral valve reconstruction, whether performed manually or robotically assisted via endoscopy, is subject to a well-publicized learning curve, so the likelihood of LCX compromise could be conflated with surgeon′s expertise (25, 26). Needle guidance and suture anchoring to the posterior leaflet require special attention and accuracy. Furthermore, due to the anatomical location of the LCX, it seems reasonable to anchor a suture no further than 3 mm away from the posterior mitral valve annulus (27). Caruso et al. therefore advocate the implantation of a flexible annuloplasty ring in the high-risk population classified by the team of authors (13). Likewise, suture anchoring of the annuloplasty ring in the transition area of the anterolateral commissure and the P1 area is also partially omitted (13), which again seems controversial (28). It is also worth noting that extensive annular resection in the posterior region can increase the risk of LCX damage in the presence of pronounced calcifications and should only be performed with the utmost attention if absolutely necessary. At best, such a maneuver is openly communicated within the team.

As already mentioned severe calcification of the mitral valve and ablation of the calcium appears to be risky, as does the use of non-undersized annuloplasty rings and surgical closure of the left atrial appendage and an often-done concomitant surgical ablation to prevent arterial embolism due to atrial fibrillation (29, 30). Unanswered but conceivable remains the question whether annular dilatation represents an independent risk. Likewise, reoperations are conceivable to increase the risk.

## 3. Diagnosis of LCX injury

The clinical presentation of LCX compromise after MV repair or replacement is heterogeneous and depends on the size and functional relevance of the LCX supply territory. Sudden deterioration of coronary blood flow due to LCX occlusion or distortion may lead to severe hemodynamic instability or recurrent malignant arrhythmias rendering weaning from cardiopulmonary bypass unsuccessful.

Yet, there is a remarkable number of patients presenting completely asymptomatic first and myocardial ischemia may only become apparent through ST segment changes or arrhythmias in the early postoperative period (3). Weaning from the CPB supported by low dose catecholamines often succeeds uneventfully, despite the occlusion of the LCX. This is explained by the different supply areas of the left and right coronary arteries. With a left coronary dominant circulation, the LCX supplies the lateral and inferior myocardial wall. In addition, the posterior interventricular coronary artery emerges from the LCX in 10% of the patients. With right coronary dominant circulation, only the lateral wall of the left ventricle is supplied by the LCX. For this reason, ECG changes are seen more frequently, especially in the inferior and lateral leads with left-dominance type, while in the right-dominance type, ECG changes are found less frequently (31).

Intraoperative detection of regional wall motion abnormalities (RWMA) pre- and post-cardiopulmonary (CPB) bypass is one of the characteristic features of perioperative transoesophageal echocardiography (TOE) (32, 33). It has been well demonstrated that appropriate expertise in recognizing regional wall motion abnormalities in the perioperative TOE can positively influence the decision-making process at an early stage (1, 33). However, clinical presentation of LCX injury following MV repair is heterogenous and intraoperative temporary RWMA are not specific for iatrogenic coronary occlusion. For example, RWMA are not uncommon in case of air embolism, primarily of the right coronary artery (RCA) for anatomical reasons (because of their ventral position) but also conceivable for all other coronaries, also presenting with electrocardiographic (ECG) changes and hemodynamic instability, which can be well echocardiographically detected (34). A right coronary air embolism usually results in a temporary right ventricular dysfunction and a consecutive left ventricular hypokinesia with relative hypovolemia (31). But the combination of ECG highly suspicious of transmural infarction and the simultaneous new onset of RWMA in TOE should always suggest an iatrogenic coronary occlusion as a differential diagnosis. In a review of 44 published LCX injuries in the context of mitral valve surgery, Hiltrop et al. reported that new or dynamic RWMA were detected in 80% of these cases by means of perioperative TOE (21).

**Figure 1:**
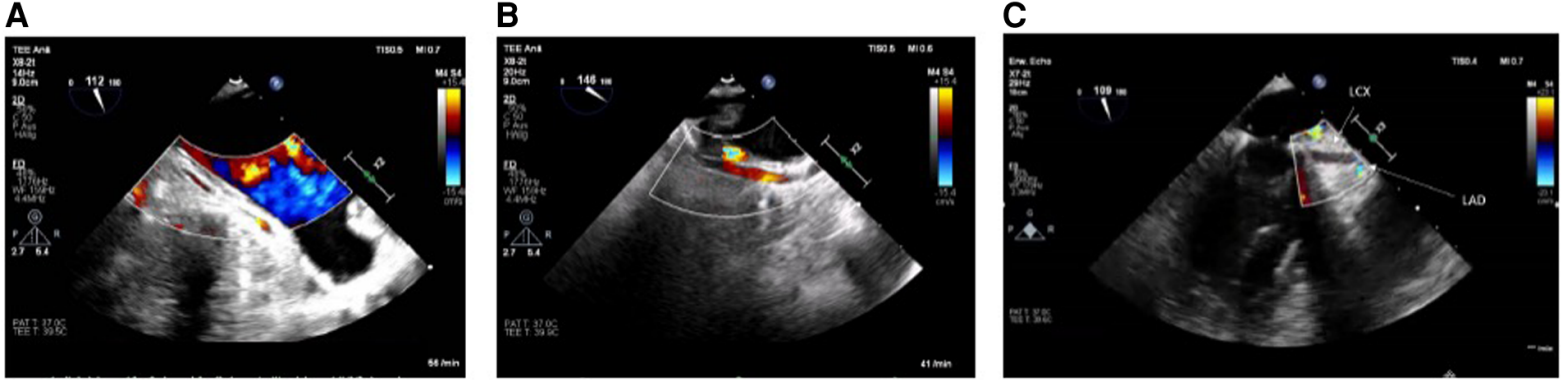
(**A**) LCX in long axis without flow in the midsegment through suture occlusion (TOE view: modified mid-esophageal aortic valve long axis view according to ender et al. (35) (ME AV LAX) with color flow Doppler, Nyquist limit <30 cm/s). (**B**) As a contrast: inconspicuous longitudinal view of a prominent LCX (TOE View: modified Mid-esophageal long axis view (mod. ME LAX) with color flow Doppler, Nyquist limit <30 cm/s. (**C**) Prominent LCX in long axis without flow detection in the proximal third and without the possibility of echocardiography imaging the LCX in the full length due to an estimated occlusion to be expected further peripherally [TOE view: modified mid-esophageal two chamber view (ME 2CH) with color flow Doppler, Nyquist limit <30 cm/s].

If no flow (in case of circumflex obstruction) or aliasing (in case of circumflex stenosis) can be visualized after mitral valve repair or if there is even an abruption seen of the LCX at best with concordant regional wall motion abnormalities (see Figures 1A–C), the findings should be communicated immediately to the surgical team and the targeted therapeutic approach should be discussed. The important role played by the attentive anesthesiologist is impressively emphasized by Landa et al. in their recently published case report in interpreting the ECG and identifying new-onset regional wall motion abnormalities as early signs of myocardial ischemia (36). Using epicardial ultrasound for vascular flow measurements is another way to detect coronary flow impairment, albeit not widely used in the clinical practice (37–40).

## 4. Therapeutic approaches

Therapeutic options consist of restoring the limited or completely occluded blood flow to the LCX supply area as soon as possible after vessel distortion. Theoretically, if the patient is still in the operating room and has not yet been antagonized with protamine, it is possible to re-establish extracorporeal circulation and reopen the left atrium. Then the surgeon could try to release the predisposing sutures that supposedly occlude the blood flow of the LCX or reposition the annuloplasty ring (41). This concept is based on the theoretical consumption that the LCX regains its previous perfusion with consecutive restoration of blood flow in the LCX after the normal anatomy has been reconstituted. The success of this maneuver should then be confirmed be disappearance of ischemic ECG changes and wall motion abnormalities in TOE.

However, this approach is limited by the fact that the underlying pathological mechanisms are more diverse. In the simplest case, a suture encircling the coronary vessel prevents blood flow, which would support the above thesis of suture loosening. Equally, however, the suture material may have passed through and perforated the coronary vessel. Also, thrombotic flow interruption, laceration of the surrounding tissue, or distortion of the tissue and vessel are possible sources of cause (1, 3, 9, 10, 15, 21). Moreover, tissue injury, especially under full anticoagulation with heparin, may result in small hematomas, which in turn may be associated with external compression of the coronary vessels. Furthermore, spasms of the coronaries are conceivable. In conclusion, the underlying etiology often cannot be delineated beyond doubt. Therefore, this approach is usually not pursued further, especially since the result of mitral valve reconstruction may also be negatively affected. In their review, Hiltrop et al. showed that in 42% of occluded LCX coronary arteries, the surgeon decided to perform direct bypass grafting, whereas 58% left the therapy to cardiologists by primary percutaneous coronary intervention (PCI) (21).

**Figure 2:**
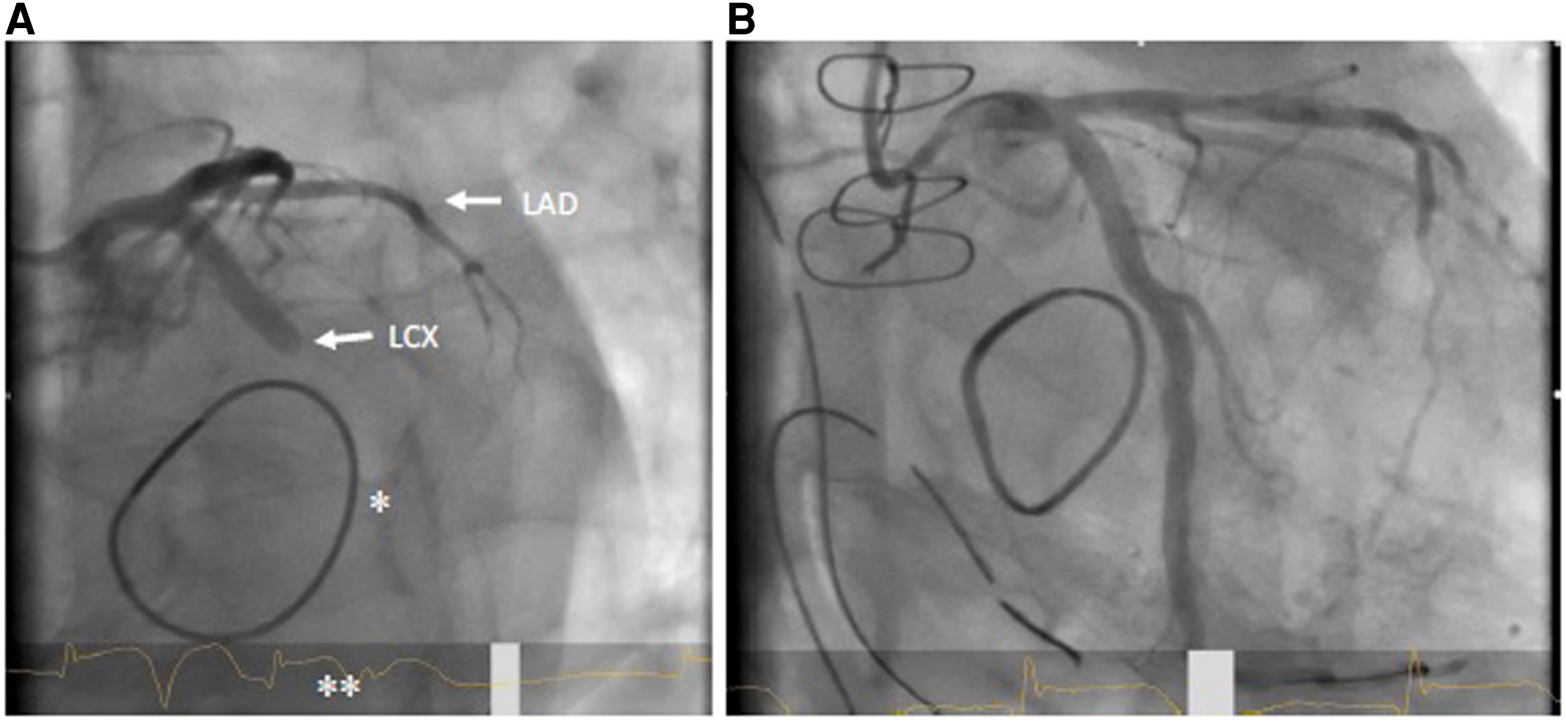
(**A**) coronary angiogram LAO 30°/caudal 20° with LCX occlusion (arrow) in the proximal to mid segment; also visible: mitral valve annuloplasty ring (*); ST elevation in ECG (**). (**B**) Flow in LCX after successful stent PCI.

Primary PCI (see Figures 2A,B) is the method of choice in case of delayed diagnosis and an alternative to immediate bypass grafting in the operating room (OR). However, failure to pass the stenosis with a guidewire in case of total occlusion renders PCI impossible and therefore demands secondary coronary artery bypass grafting (CABG). Moreover, patients undergoing PCI for recanalization of LCX require immediate dual antiplatelet therapy, which in some circumstances may increase the risk of postoperative hemorrhage and consecutive resternotomy, as well as consecutive blood product consumption. On the other hand, CABG to the posterior wall of the left ventricle is usually associated with a need of full sternotomy. In most cases, a venous graft will perhaps be preferred over an arterial graft due to time constraints, as a mammary graft preparation is likely to take longer. Venous grafting is also more likely to be preferred if some time has already elapsed due to unsuccessful PCI and the patient needs to be emergently taken back to the OR. In any case of surgical solution of coronary artery occlusion, it should be kept in mind that in these patients excessive lifting of the heart may provoke atrioventricular rupture, which is deleterious with high probability (42, 43). This is important in the presence of calcification of the annulus and an application of a full annuloplasty (44). Furthermore, it must also be considered that unstable patients are probably less suitable for PCI, since the patient usually must be transferred to a cardiac catheterization laboratory first. The transport itself represents an independent risk factor. Nevertheless, if a decision is made to perform a PCI instead of CABG, the fact that this can also be performed in a hybrid room, if necessary even before thoracic closure, should be considered to provide the best available and individualized therapy (39).

## 5. Discussion

Any preoperative imaging, depending on its spatial resolution, can be used to evaluate the distance between LCX and mitral valve annulus. Preoperative multidetector coronary computed tomography (MD-CT) is of special interest, because stenoses and wall irregularities can be reproducibly detected as predilection sites, especially in the proximal LCX (45). Furthermore, coronary CT is safer than cardiac catheterization and offers the possibility not to stumble upon coronary anomalies (46).

Some authors advocate to routinely perform a preoperative cardiac catheterization before MV surgery, not only to exclude significant coronary heart disease, but also to identify patients at high risk for LCX injury during MV surgery (2, 5, 47). But, as outlined above, recently published studies on coronary anatomy were not able to show a clear correlation between coronary dominance pattern and risk of LCx injury during MV surgery. This could be partly due to confounding factors, such as size of the LCX diameter or the underlying etiology of mitral regurgitation. Risk identification by preoperative invasive coronary angiography therefore seems to be unreliable, even if a left dominant pattern or a large LCX appears to be a predisposing factor for perioperative LCx occlusion. Nevertheless, coronary anatomy and the predominant coronary circulation pattern should be always discussed as a part of the preoperative team time-out. Various studies identified the area close to P1 segment to be at highest risk for LCX injury, partly independent of the coronary circulation pattern (13, 21, 48, 49). The LCx course near to the P1 segment can, however, often easily visualized by means of transesophageal echocardiography (14, 25). A possible prevention and risk stratification strategy could therefore include the measurement of the distance from the MV annulus to the LCX (CAD) during routine preoperative TOE in patients where the CAD is not already known from preoperative MD-CT (26). At best, the LCX is visualized in the short and long axis with and without color Doppler. After weaning from extracorporeal circulation, this is repeated and any iatrogenic LCX impairment can be immediately inferred. Likewise, existing regional wall motion abnormalities should be detected and noted to be able to differentiate them from newly appeared RWMA. It is possible that fusion imaging offers a new approach to prevention by combining synergistic information from multiple imaging modalities (50).

In the future, hybrid operating rooms may further facilitate finding the optimal therapeutic approach in case of iatrogenic LCX injury during MV surgery, while the patient transfer to the cath-lab will no longer be necessary. This enables the surgical team to find the best treatment strategy depending on the etiology causing the circumflex injury in a timely manner.
